# Adherence to nine-month isoniazid for latent tuberculosis infection in healthcare workers: a prospective study in a tertiary hospital

**DOI:** 10.1038/s41598-020-63156-8

**Published:** 2020-04-15

**Authors:** Sung Jun Chung, Hyun Lee, Gun Woo Koo, Ji-Hee Min, Yoomi Yeo, Dong Won Park, Tai Sun Park, Ji-Yong Moon, Sang-Heon Kim, Tae Hyung Kim, Jang Won Sohn, Ho Joo Yoon

**Affiliations:** Department of Internal Medicine, Hanyang University College of Medicine, Seoul, Korea

**Keywords:** Tuberculosis, Outcomes research

## Abstract

Poor adherence to medication can lead to treatment failure in healthcare workers (HWCs) with latent tuberculosis infection (LTBI) who are at high risk of developing active tuberculosis. However, the factors associated with non-completion of nine-month LTBI treatment with isoniazid (9 H) have not been well studied. We investigated the completion rate and factors affecting adherence to LTBI treatment with 9 H among HCWs. A prospective cohort study of 114 HCWs who were diagnosed with LTBI by QuantiFERON-TB Gold In-Tube tests were performed in a single university hospital between June 2016 and December 2017. All patients received the 9 H LTBI treatment. At each visit, treatment adherence and development of adverse reactions to isoniazid were evaluated via a standard questionnaire. To evaluate the impact of the severity of hepatotoxicity on non-completion of LTBI treatment, we classified hepatotoxicity into two groups: severe hepatotoxicity was defined as alanine aminotransferase >3.0 times the upper normal limit (UNL) with symptoms or  = 5.0 times the UNL. Mild hepatotoxicity was defined as alanine aminotransferase>UNL, but not meet the definition of severe hepatotoxicity. Overall, 71 HCWs (62.3%) completed LTBI treatment with 9 H while 43 HCWs (37.7%) discontinued their treatment. Most discontinuation (81.4%, 35/43) occurred during the first three months of treatment. There were no significant differences in age, sex, occupation, or comorbidities between the HCWs who completed and those who discontinued LTBI treatment. However, HCWs who discontinued LTBI treatment had more hepatotoxicity than those who completed treatment (44.2% vs. 11.3%, *P* < 0.001). Cox proportional hazard analysis revealed that hepatotoxicity is the only factor significantly associated with discontinuation of 9 H LTBI treatment (unadjusted HR = 2.89, 95% CI = 1.62–5.46). In multivariable analysis, not only severe hepatotoxicity (adjusted HR = 7.99, 95% CI = 3.05–20.94) but also mild hepatotoxicity was significantly associated with discontinuation of LTBI treatment (adjusted HR = 2.34, 95% CI = 1.05–5.21). The completion rate of 9 H LTBI treatment was 62.3% among HCWs. While age, sex, occupation, and pretreatment comorbidities were not associated with treatment completion, isoniazid-induced hepatotoxicity significantly affected adherence.

## Introduction

Healthcare workers (HCWs) are vulnerable to *Mycobacterium tuberculosis* infection due to a higher risk of exposure to patients with active pulmonary tuberculosis^1–3^. Latent tuberculosis infection (LTBI) among HCWs has been reported to be as high as 64%, worldwide^4,5^. Three studies in Korea reported that LTBI prevalence was about 15%–17% among HCWs^3,6,7^. In our previous study, about one-third of HCWs were diagnosed with LTBI by interferon-gamma releasing assay (IGRA), which was higher than reported by other studies^8^.

Successfully treating HCWs for LTBI is crucial for tuberculosis (TB)-control because HCWs who develop active pulmonary TB can easily transmit their infections to patients. Across communities, the adherence rate for LTBI treatment is unsatisfactory, ranging from 44%–81% in the general population^9–15^ and 17%–100% among HCWs^7,12,14,16–19^. However, studies that evaluated LTBI adherence rates among HCWs were limited by their retrospective study designs, variable treatment regimens, and relatively small subject populations, all of which could explain the wide range of completion rates observed.

The currently recommended LTBI treatment regimens according to the American Thoracic Society (ATS), the Centers for Disease Control and Prevention (CDC), Infectious Diseases Society of America (IDSA), and the World Health Organization (WHO) include nine-month treatment with isoniazid (9 H), four-month treatment with rifampin (4 R), or three-month treatment with a combination of isoniazid and rifampin (3HR), with a broad preference for the 9 H regimen^20,21^. The Korean guidelines for LTBI treatment also adopted these recommendations^22^. Accordingly, the 9 H regimen is widely used in clinical practice as the preferred treatment. However, given that it is the longest treatment regimen, there is concern that it has an inferior completion rate compared with other regimens^13^. However, few studies have prospectively evaluated this concern among HCWs.

Thus, we prospectively evaluated the completion rate and factors associated with adherence to the 9 H regimen among HCWs with LTBI who were diagnosed by IGRA.

## Methods

### Study design and population

**Figure 1:**
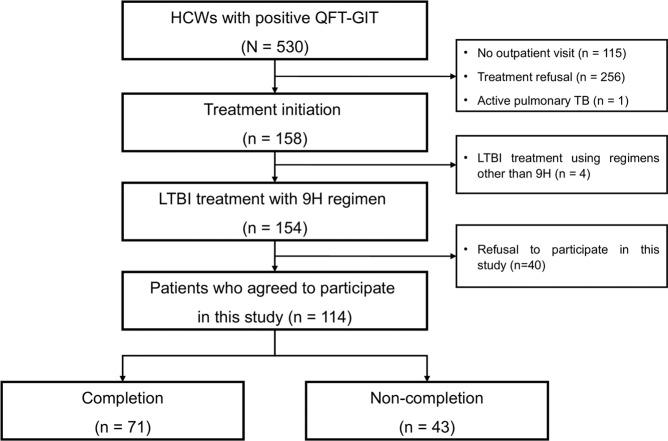
Flow chart of LTBI treatment in HCWs who were diagnosed with LTBI. *LTBI* latent tuberculosis infection, *HCW* healthcare worker, *QFT-GIT* QuantiFERON-TB Gold In-Tube tests, *OPD* outpatient department, *9 H* 9-month treatment with isoniazid.

A prospective cohort study to assess completion rate and factors affecting adherence to LTBI treatment among HCWs was performed in a single university hospital between June 2016 and December 2017. During the study period, QuantiFERON-TB Gold In-Tube tests (QFT-GIT; Cellestis Ltd., Carnegie, VIC, Australia) were performed for 2,379 HCWs; 530 HCWs were positive according to QFT-GIT and 158 HCWs visited an outpatient clinic and agreed to begin LTBI treatment. After excluding four HCWs who received a treatment regimen other than 9 H and 40 HCWs who refused to participate in this study, a total of 114 HCWs participated in this study (Fig. 1). Data on 86 patients who agreed to participate in this study were previously reported^8^.

### Ethics approval and consent to participate

Written informed consent was obtained from each HCW who participated in this study. The study protocol was approved by the Institutional Review Board of Hanyang University Hospital (IRB number 2017-02-018). All methods were performed in accordance with the relevant guidelines and regulations.

### LTBI treatment protocol, follow-up, and monitoring of adverse reactions

All HCWs who agreed to LTBI treatment received isoniazid at a dose of 5 mg/kg up to 300 mg once daily for nine months (270 doses)^21^. Generally, participants were followed-up at two-week intervals for one month, followed by one-month intervals until treatment completion. At each visit, participants were asked to report any adverse events via a structured form. All suspected adverse reactions were investigated, managed, and reported according to standardized in-hospital protocol. During the 9 H treatment, patients reported all their remaining drug doses to every visit for pill counts.

Using a standard questionnaire, brief physical assessment, and routine laboratory examination, patients were evaluated to check for the following adverse events: gastrointestinal symptoms, dermatologic symptoms and signs, myelosuppression, hepatotoxicity, peripheral neuropathy, and other symptoms. To evaluate the impact of the severity of hepatotoxicity on non-completion of LTBI treatment, we classified hepatotoxicity into two groups: severe hepatotoxicity was defined as alanine aminotransferase >3.0 times the upper normal limit (UNL) with symptoms or >5.0 times the UNL, according to the American Thoracic Society guideline^23^ and Korean guideline^22^. Mild hepatotoxicity was defined as alanine aminotransferase >UNL, but not meet the definition of severe hepatotoxicity. Gastrointestinal symptoms included nausea, anorexia, vomiting, and diarrhea. Dermatological symptoms and signs included itching, urticaria, or rash. Myelosuppression was defined as neutropenia (absolute neutrophil count <1,000/mm^3^), thrombocytopenia (platelet < 100,000/mm^3^), or anemia (Hb ≤ 10.5 g/dL). Other symptoms included headache, dizziness, general weakness, fatigue, or fever.

### Outcomes

The primary outcomes of interest were completion rates and factors associated with non-completion among HCWs who received the 9 H regimen for LTBI treatment. Treatment completion was defined as receipt of ≥80% of isoniazid doses within nine months of treatment initiation^13,15^.

### Statistical analyses

Categorical variables are presented as numbers with percentages, and continuous variables are presented as medians with interquartile ranges (IQR) or means with standard deviations, as appropriate. Pearson’s chi-square test or Fisher’s exact test were used to comparing categorical variables. Student’s t-test or the Mann-Whitney U test was used to compare continuous variables. We used the Kaplan-Meier method to estimate the proportion of HCWs who discontinued LTBI treatment during follow-up.

To evaluate the factors affecting LTBI treatment adherence, univariable and multivariable Cox proportional hazard analysis was performed. Factors included in the Cox models included age, sex, body mass index, hypertension, chronic liver disease, work group (patient-related HCWs vs. patient-unrelated HCWs), hepatotoxicity, gastrointestinal symptoms, dermatologic symptoms, hematologic symptoms, peripheral neuropathy, and other adverse reactions. All tests were two-tailed, and a *P*-value <0.05 was considered statistically significant. All statistical analyses were performed using the Statistical Package for the Social Sciences (SPSS) for Windows (version 24.0; IBM Corp., Armonk, NY, USA) and STATA (version 15.0; Stata Corporation, College Station, TX, USA).

## Results

### Study subjects

The baseline characteristics of the 114 subjects who initiated 9 H treatment are summarized in Table 1. The median age was 42 years, and 69.3% (n = 79) were female. The median body mass index was 23.1 kg/m^2^. Subjects included 89 patient-related HCWs (78.1%) and 25 patient-unrelated HCWs (21.9%). The common comorbidities were hypertension (n = 10, 8.8%), chronic liver disease (n = 4, 3.5%), diabetes mellitus (n = 2, 1.8%), and chronic kidney disease (n = 1, 0.9%). None of the HCWs tested positive for human immunodeficiency virus infection.

**Table 1:** Baseline characteristics of the study population.

	Total (N = 114)
Age, years	42 (32–52)
Sex, female	79 (69.3)
BMI, Kg/m^2^	23.1 (19.6–26.6)
Occupation	
Patient-related HCWs	89 (78.1)
Physicians	11/89 (12.4)
Nurses	54/89 (60.7)
Others	24/89 (26.9)
Patient-unrelated HCWs	25 (21.9)
Comorbidity	
Hypertension	10 (8.8)
Chronic liver disease	4 (3.5)
Diabetes mellitus	2 (1.8)
Chronic kidney disease	1 (0.9)

Data are presented as either medians and interquartile ranges or as numbers and percentages, as appropriate.

^†^Patient-unrelated HCWs included administrative staff, cooks, and cleaning staff.

*BMI* body mass index, *HCW* healthcare worker.

### Completion rate

As shown in Table 2, while 62.3% of HCWs (71/114) completed LTBI treatment, 37.7% (43/114) discontinued LTBI treatment. The completion proportions were 54.5% (12/22) in the twenties, 56.3% (9/16) in the thirties, 66.7% (34/51) in the forties, and 64.0% (16/25) in the fifties, respectively. By sex, the completion proportions were 62.9% among men and 62.0% among women. The proportions of completion by occupation were 54.5% (6/11) among doctors, 63.0% (34/54) among nurses, 62.5% (15/24) among other patient-related HCWs, and 64.0% (16/25) among patient-unrelated HCWs.

**Table 2:** Comparison of the clinical characteristics of HCWs who completed versus those who discontinued 9-month isoniazid treatment.

	Total (N = 114)	Completion (n = 71)	Non-completion (n = 43)
Age, years	44 (34–49)	44 (34–54)	43 (33–53)
Twenties	22 (19.3)	12/22 (54.5)	10/22 (45.5)
Thirties	16 (14.0)	9/16 (56.3)	7/16 (43.7)
Forties	51 (44.7)	34/5 (66.7)	17/51 (33.3)
Fifties	25 (21.9)	16/25 (64.0)	9/25 (36.0)
Sex			
Male	35 (30.7)	22/35 (62.9)	13/35 (37.1)
Female	79 (69.3)	49/79 (62.0)	30/79 (38.0)
Occupation			
Patient-related HCWs	89 (78.1)	55/89 (61.8)	34/89 (38.2)
Doctors	11 (15.5)	6/11 (54.5)	5/11 (45.5)
Nurses	54 (47.4)	34/54 (63.0)	20/54 (37.0)
Others	24 (21.1)	15/24 (62.5)	9/24 (37.5)
Patient-unrelated HCWs	25 (21.9)	16/25 (64.0)	9/25 (36.0)
Comorbidity			
Hypertension	10 (8.8)	8/10 (80.0)	2/10 (20)
Chronic liver disease	4 (3.5)	2/4 (50.0)	2/4 (50)
Diabetes mellitus	2 (1.8)	2/2 (100.0)	0/2 (0)
Chronic kidney disease	1 (0.9)	1/1 (100.0)	0/1 (0)

Data are presented as either medians and interquartile ranges or as numbers and percentages, as appropriate.

^*^Others included radiology technicians, laboratory technicians, physical therapists, and medical technicians.

^†^Patient-unrelated HCWs included administrative staff, cooks, and cleaning staff.

*HCW* healthcare worker, *LTBI* latent tuberculosis infection.

**Figure 2:**
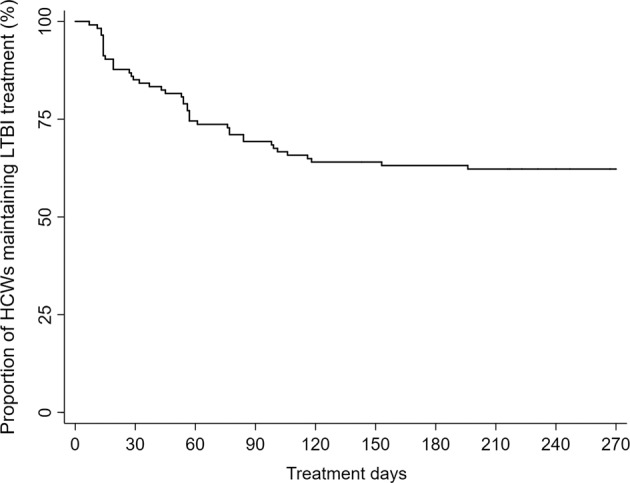
The proportion of HCWs on LTBI treatment with 9 H over the study period. *HCW* healthcare worker, *9 H*, 9-month treatment with isoniazid.

As shown in Fig. 2, during the first three months of treatment duration, 35 patients (30.7%) discontinued treatment, and a total of 41 patients (36.0%) discontinued treatment by month six.

### Factors affecting non-completion

As shown in Table 3, the overall adverse reaction rate was 61.4% (70/114), which includes 57.7% of HCWs who completed and 67.4% of HCWs who discontinued LTBI treatment (*P* = 0.422). As shown in Table 3, there were no significant differences in the rates of adverse reactions, including gastrointestinal (38.0% vs. 27.9%; *P* = 0.350), dermatologic (11.3% vs. 16.3%; *P* = 0.129), hematologic (18.3% vs. 18.6%; *P* = 0.250), peripheral neuropathy (2.8% vs. 9.3%; *P* = 0.133), and others (36.6% vs. 30.2%; *P* = 0.523), between HCWs who completed and those who discontinued LTBI treatment. In contrast, hepatotoxicity was significantly higher among HCWs who discontinued compared with those who completed LTBI treatment (44.2% vs. 11.3%, *P* < 0.001). Of the 27 HCWs who had hepatotoxicity, 19 (70.4%) discontinued LTBI treatment, about two-thirds of whom developed hepatotoxicity within three months of LTBI treatment initiation. None of the HCWs died from adverse reactions.

**Table 3:** Adverse reactions to LTBI treatment with 9-month treatment with isoniazid.

	Total (N = 114)	Completion (n = 71)	Non-completion (n = 43)	*P*
Any adverse reactions	70 (61.4)	41(57.7)	29 (67.4)	0.422
Gastrointestinal	39 (34.2)	27 (38.0)	12 (27.9)	0.350
Nausea	27 (23.7)	16 (22.5)	11 (25.6)	
Anorexia	8 (7.0)	6 (8.5)	2 (4.7)	
Diarrhea	2 (1.8)	2 (2.8)	0 (0)	
Skin	15 (13.2)	8 (11.3)	7 (16.3)	0.129
Itching	12 (10.5)	6 (8.5)	6 (14.0)	
Urticaria	4 (3.5)	1 (1.4)	3 (7.0)	
Eczema	4 (3.5)	3 (4.2)	1 (2.3)	
Hematologic	21 (18.4)	13 (18.3)	8 (18.6)	0.250
Neutropenia	6 (5.3)	3 (4.2)	3 (7.0)	
Anemia	12 (10.5)	7 (9.9)	5 (11.6)	
Thrombocytopenia	4 (3.5)	3 (4.2)	1 (2.3)	
Hepatotoxicity^*^	27 (23.7)	8 (11.3)	19 (44.2)	<0.001
Mild hepatotoxicity	17 (14.9)	7 (9.9)	10 (23.3)	
Severe hepatotoxicity	10 (8.8)	1 (1.4)	9 (20.9)	
Peripheral neuropathy	6 (5.3)	2 (2.8)	4 (9.3)	0.133
Others	39 (34.2)	26 (36.6)	13 (30.2)	0.523
Headache	10 (8.8)	7 (9.9)	3 (7.0)	
Dizziness	12 (10.5)	7 (9.9)	5 (11.6)	
General weakness	31 (27.2)	21 (29.6)	10 (23.3)	
Fever	4 (3.5)	2 (2.8)	2 (4.7)	

The data are presented as numbers and percentages.

^*^Mild hepatotoxicity was defined as ALT > UNL, but not meet the definition of severe hepatotoxicity. Severe hepatotoxicity was defined as ALT > 3.0 times UNL with symptoms or >5.0 times UNL without symptoms.

*ALT* alanine aminotransferase, *UNL* upper normal limit.

Next, to determine significant factors contributing to non-completion of LTBI treatment, univariable and multivariable Cox proportional regression analyses were performed. Hepatotoxicity was the only clinical factor that significantly affected the non-completion of LTBI among HCWs who initiated LTBI treatment. Compared with HCWs without hepatotoxicity, those with mild hepatotoxicity and severe hepatotoxicity were 2.34 (95% CI, 1.05–5.21) and 7.99 (95% CI, 3.05–20.94) times more likely to discontinue LTBI treatment, respectively (Table 4).

**Table 4:** Cox proportional hazard analysis for factors affecting non-completion during LTBI treatment among HCWs.

	Univariable analysis		Multivariable analysis^‡^	
	Hazard ratio (95% CI)	*P*	Hazard ratio (95% CI)	*P*
Age group	0.99 (0.96–1.01)	0.314		
The twenties	Reference		Reference	
Thirties	0.93 (0.35–2.45)	0.890	0.99 (0.32–3.10)	0.983
Forties	0.73 (0.34–1.59)	0.432	0.70 (0.27–1.87)	0.480
Fifties	0.67 (0.26–1.70)	0.401	0.43 (0.16–1.22)	0.113
Sex, male	1.06 (0.55–2.03)	0.863	1.08 (0.48–2.44)	0.856
BMI, kg/m^2^	0.99 (0.90–1.08)	0.783	1.01 (0.91–1.12)	0.873
Work group				
Patient-related HCWs	Reference		Reference	
Patient-unrelated HCWs	1.13 (0.54–2.36)	0.742	0.68 (0.28–1.63)	0.384
Comorbidity				
Hypertension	2.02 (0.49–8.36)	0.331	0.78 (0.17–3.55)	0.746
Chronic liver disease	0.78 (0.19–3.21)	0.726	0.79 (0.13–4.94)	0.803
Adverse reactions	1.11 (0.50–2.49)	0.860	—	
Hepatotoxicity^*^	2.89 (1.62–5.46)	<0.001	—	
No hepatotoxicity	Reference		Reference	
Mild hepatotoxicity	2.26 (1.08–4.74)	0.030	2.34 (1.05–5.21)	0.038
Severe hepatotoxicity	4.71 (2.14–10.34)	<0.001	7.99 (3.05–20.94)	<0.001
Gastrointestinal	0.85 (0.45–1.60)	0.607	0.77 (0.37–1.62)	0.491
Dermatologic	1.52 (0.73–3.16)	0.266	2.25 (0.91–5.55)	0.080
Hematologic	0.64 (0.25–1.62)	0.344	0.69 (0.26–1.85)	0.463
Peripheral neuropathy	2.24 (0.80–6.27)	0.125	3.23 (0.83–12.51)	0.090
Others^†^	0.90 (0.48–1.67)	0.738	0.98 (0.51–1.88)	0.945

Data are presented as ratios and 95% CIs.

^*^Mild hepatotoxicity was defined as ALT > UNL, but not meet the definition of severe hepatoxicity. Severe hepatotoxicity was defined as ALT > 3.0 times UNL with symptoms or >5.0 times UNL without symptoms.

^†^Others include headache, dizziness, general weakness, fatigue, and fever.

^‡^Adjusted for age group, sex, BMI, work group, comorbidity (hypertension and chronic liver disease), and each type of adverse events (the severity of hepatotoxicity, gastrointestinal, dermatologic, and hematologic events, peripheral neuropathy, and others).

*CI* confidence interval, *BMI* body mass index, *HCW* healthcare worker, *UNL* upper normal limit.

## Discussion

To the best of our knowledge, this is the first prospective observational study to evaluate completion rates and factors associated with poor adherence to the 9 H LTBI treatment among HCWs. We found that about two-thirds of HCWs who initiated LTBI treatment completed LTBI treatment and one-third discontinued LTBI treatment. Most discontinuation occurred within three months of LTBI treatment initiation, and hepatotoxicity was the only significant factor associated with non-completion of LTBI treatment. We further showed even mild hepatotoxicity, not meeting the definition of hepatotoxicity of the current guideline, was also associated with discontinuation of LTBI treatment.

The completion rate for the 9 H LTBI treatment among HCWs in this study was 62.3%, which is higher than that reported in a previous study (58%)^19^. Regarding completion rates for other LTBI treatment regimens among HCWs, approximately 70% and 80% of adherence rates were reported with 4R^16,19^, 75% and 82% with a six-month isoniazid regimen^12,18^, and 61.7% and 100% with 3HR^7,16^. These results indicate that completion rates for the 9 H regimen among HCWs is relatively lower than those for shorter treatment regimens. However, our study results also show that approximately 80% of HCWs who did not complete LTBI treatment discontinued within three months of their initial treatment, suggesting that the completion rate would not have been better with a shorter regimen (e.g., four–six-month regimen) and, thus, management of modifiable factors is especially important for improving the LTBI treatment completion rate. In addition, no randomized controlled trials that compare completion rates for the 9 H regimen with shorter regimens have been conducted in HCWs. Thus, a well-designed randomized controlled trial is needed.

In our study, the overall rate of adverse reactions was 61%, and hepatotoxicity occurred in 24% of HCWs received the 9 H regimen. A previous study found that about 41% of HCWs who received the 9 H regimen experienced adverse reactions, and 28% had hepatotoxicity^19^. The reasons for the relatively lower prevalence of hepatotoxicity despite higher overall adverse reactions in our study relative to the previous study are unclear. The prospective design of our study might be associated with higher adverse reactions because we assessed adverse reactions at one-month intervals. Thus, it is likely that we recorded more mild adverse events, especially considering that most patients, except for those experiencing hepatotoxicity, did not discontinue due to adverse reactions. However, hepatotoxicity prevalence in our study and the previous study might not be directly comparable because the other study did not clearly define their diagnostic criteria for hepatotoxicity. Regarding adverse reactions associated with other regimens, 42% and 49% of HCWs experienced adverse reactions to 4R^16,19^, 47% to 3HR^16^, and 73% to a combination of rifapentine and isoniazid^19^. However, because the definitions for adverse events might vary between studies, further studies comparing adverse reactions to various LTBI treatment regimens in HCWs are needed.

Herein, we found that hepatotoxicity was the most significant factor associated with non-completion of LTBI treatment. Of the patients who discontinued LTBI treatment, about 23% and 20% developed mild and severe hepatotoxicity, respectively. When we analyzed discontinuation rate among the patients with hepatotoxicity, surprisingly, not only those with severe hepatotoxicity in whom 90% discontinued LTBI treatment but also about 60% of those with mild hepatotoxicity discontinued LTBI treatment. The reasons for this phenomenon are not clear. It seems that the threshold for discontinuation of LTBI treatment due to hepatotoxicity might be lower than that of active pulmonary TB treatment, as the purpose of LTBI treatment is not to treat active disease but to prevent future disease. From this view, our study has provided valuable information that even mild hepatotoxicity can be a barrier to successful LTBI treatment. Accordingly, our results suggest that augmented early management of LTBI treatment adherence with a specific focus on hepatotoxicity management is very important.

This study had several limitations. First, our study was conducted in a single hospital. Because many factors can affect treatment adherence among HCWs, including LTBI prevalence, the occurrence of adverse reactions, and variation in associated stigmas, can affect the adherence of LTBI treatment in different HCWs, our findings might not be generalizable to other hospitals or countries. Second, only a minority of HCWs diagnosed with LTBI presented for treatment. Therefore, our study might have overestimated the completion rate by pre-selecting individuals inclined to seek treatment. Third, as we did not evaluate the reasons for follow-up loss, we could not identify specific reasons for discontinuation of LTBI treatment in these HCWs.

## Conclusions

In conclusion, in our study, the completion rate was 62.3% with LTBI treatment with 9 H among HCWs. Most HCWs discontinued LTBI during the first 3months after treatment initiation. Of the various adverse events, hepatotoxicity was the only significant factor associated with discontinuation. The strategies to enhance adherence during the early phage of LTBI treatment and managing hepatotoxicity properly are needed for the successful management of LTBI treatment among HCWs who receive 9 H.

## Data Availability

The data and analytic methods are available upon request.
